# Prevalence and Risk Factors of Thyroid Dysfunction in Older Adults in the Community

**DOI:** 10.1038/s41598-019-49540-z

**Published:** 2019-09-11

**Authors:** Nermin Diab, Natalie R. Daya, Stephen P. Juraschek, Seth S. Martin, John W. McEvoy, Ulla T. Schultheiß, Anna Köttgen, Elizabeth Selvin

**Affiliations:** 1 Department of Epidemiology, Bloomberg School of Public Health, Johns Hopkins University, Baltimore, MD USA; 2 Welch Center for Prevention, Epidemiology and Clinical Research, Johns Hopkins University, Baltimore, MD USA; 3 Division of General Medicine, Beth Israel Deaconess Medical Centre/Harvard Medical School, Boston, MA USA; 4 Johns Hopkins Ciccarone Center for the Prevention of Heart Disease, Division of Cardiology, Department of Medicine, Johns Hopkins University School of Medicine, Baltimore, MD USA; 5 School of Medicine, National University of Ireland, Galway Campus, Ireland; 6 National Institute for Preventive Cardiology, Galway, Ireland; 7 Institute of Genetic Epidemiology, Medical Center - University of Freiburg and Faculty of Medicine, Freiburg, Germany; 8 Renal Division, Department of Medicine IV, Medical Center - University of Freiburg Faculty of Medicine, Freiburg, Germany

**Keywords:** Risk factors, Endocrine system and metabolic diseases

## Abstract

Prevalence estimates and evidence informing treatment targets for thyroid dysfunction largely come from studies of middle-aged adults. We conducted a cross-sectional analysis to determine the prevalence of thyroid dysfunction and risk factors for abnormal thyroid tests in participants aged ≥65 in the Atherosclerosis Risk in Communities (ARIC) study (N = 5,392). We measured serum concentrations of triiodothyronine (T3), free thyroxine (FT4), thyroid peroxidase antibody (Anti-TPO), and thyroid stimulating hormone (TSH). In this population (58% women, 22% black), 17% reported medication use for thyroid dysfunction. Among those not on treatment, the prevalence of overt and subclinical hypothyroidism was 0.82% and 6.06%, respectively. Overt and subclinical hyperthyroidism affected 0.26% and 0.78%, respectively. Multivariable adjusted TSH, FT4 and T3 levels were 25%, 1.3% and 3.9% lower in blacks compared to whites, respectively. Men were less likely to be anti-TPO positive compared to women (p < 0.001). Former and never smoking were associated with lower T3 and FT4 levels compared to current smoking. The prevalence of thyroid dysfunction in older adults is nearly 25%. Multiple illnesses can interact to contribute to declines in health. Additional attention to thyroid dysfunction and screening in this age group is recommended.

## Introduction

Thyroid dysfunction is one of the most common endocrine disorders seen in clinical practice. The prevalence of thyroid dysfunction varies by age, sex, race/ethnicity, and geographically through variations in dietary iodine intake^1,2^. Abnormal thyroid function has important ramifications on health outcomes pertinent to older adults, including cardiovascular arrhythmia, metabolism, bone health, and mental health^3–7^.

Current estimates of the prevalence of thyroid dysfunction are largely derived from data in predominantly white middle-aged populations^8–10^. There are limited available data on the prevalence of thyroid dysfunction in older adults. Furthermore, some studies have shown that the TSH distribution shifts to higher values with increasing age suggesting that a universal treatment target may not be appropriate for all ages^11,12^. Currently, in clinical practice, guidelines recommend the same cut-points for thyroid hormones irrespective of age, race, or sex^13,14^.

Little is known about risk factor associations for thyroid dysfunction and correlates of the individual thyroid hormones in an older adult population. While multiple studies have examined associations of race, sex, BMI, dyslipidemia, heart rate, glycemic control, kidney function and smoking with thyroid hormone levels in middle-aged cohorts^6,9,10,12,15–18^, few studies have addressed these associations exclusively in an older adult population^19,20^.

In light of the above, the objectives of this study were to (1) investigate the prevalence of thyroid dysfunction in an older community-based U.S. population, (2) better understand demographic and clinical risk factors for thyroid dysfunction in this older age group, and (3) examine the relationship between serum concentrations of triiodothyronine (T3), free thyroxine (FT4), thyroid peroxidase antibody (anti-TPO), and thyroid stimulating hormone (TSH) with demographic and clinical risk factors.

## Results

Among the 5,392 participants, using the ARIC defined (or Roche defined) cut-offs, the prevalence of hypothyroidism was 23.78% (28.90%) and the prevalence of hyperthyroidism was 1.15% (0.36%). Among those with hypothyroidism and hyperthyroidism, 71.06% (56.13%) and 9.68% (30%), respectively, were treated previously. The prevalence of overt hypothyroidism in untreated participants was 0.82% (2.21%), subclinical hypothyroidism was 6.06% (10.5%), overt hyperthyroidism was 0.26% (0.07%), subclinical hyperthyroidism was 0.78% (0.18%) and euthyroidism was 75.07% (70.74%). The prevalence of participants with treated hypothyroidism or hyperthyroidism was 16.90% (16.22%) and 0.11% (0.11%) respectively (Table 1). Significant differences in prevalence of thyroid dysfunction were observed across participant characteristics. Women had a higher prevalence of treated hypothyroidism compared to males (P < 0.001). Subclinical hypothyroidism and treated hypothyroidism were significantly greater in whites compared to blacks (P < 0.001), while prevalence of subclinical hyperthyroidism was greater in blacks compared to whites (P = 0.002). Higher age and BMI were significantly associated with higher prevalence of subclinical hypothyroidism (P = 0.008 and 0.004 respectively) and treated hypothyroidism (P = 0.001 and 0.049 respectively) (Table 1).

**Table 1:** Prevalence of thyroid dysfunction, ARIC Visit 5 (2011–2013).

	Euthyroid	Overt Hypothyroidism	Subclinical Hypothyroidism	Overt Hyperthyroidism	Subclinical Hyperthyroidism	Treated Hypothyroidism	Treated Hyperthroidism
	N = 4048	N = 44	N = 327	N = 14	N = 42	N = 911	N = 6
Prevalence (%)	75.07	0.82	6.06	0.26	0.78	16.90	0.11
Sex (%)							
Male	82.94	1.06	6.51	0.22	0.75	8.37	0.13
Female	69.41	0.64	5.74	0.29	0.80	23.03	0.10
P-value*	—	0.272	0.652	0.430	0.456	**<0.001**	0.854
Race (%)							
Black	79.83	1.12	3.43	0.34	1.55	13.65	0.09
White	73.76	0.73	6.79	0.24	0.57	17.79	0.12
P-value	—	0.303	**<0.001**	0.619	**0.002**	**<0.001**	0.714
Age (%)							
Mean ± SD	75.24 [±5.01]	76.73 [±6.00]	76.43 [±5.18]	75.86 [±4.82]	75.36 [±5.29]	75.98 [±5.30]	74.83 [±5.49]
65–70	78.09	0.79	5.01	0.20	0.59	15.13	0.20
71–75	77.06	0.56	5.37	0.36	1.02	15.53	0.10
76–80	74.02	0.84	6.74	0.14	0.49	17.77	0.00
80+	69.59	1.31	7.55	0.30	0.91	20.14	0.20
P-value	—	0.123	**0.008**	0.635	0.299	**0.001**	0.379
BMI(kg/m^2^) (%)							
Mean ± SD	28.66 [±5.57]	29.60 [±4.76]	27.65 [±5.17]	28.26 [±6.97]	29.06 [±4.85]	29.32 [±6.19]	23.00 [±2.38]
<25.0	75.41	0.65	7.61	0.36	0.57	15.07	0.29
25.0–29.9	75.20	0.79	6.31	0.28	0.83	16.47	0.09
30.0+	74.68	0.97	4.61	0.16	0.86	18.75	0.00
P-value	—	0.585	**0.004**	0.542	0.602	**0.049**	0.050

*P values were calculated using Chi-square analysis.

**Figure 1:**
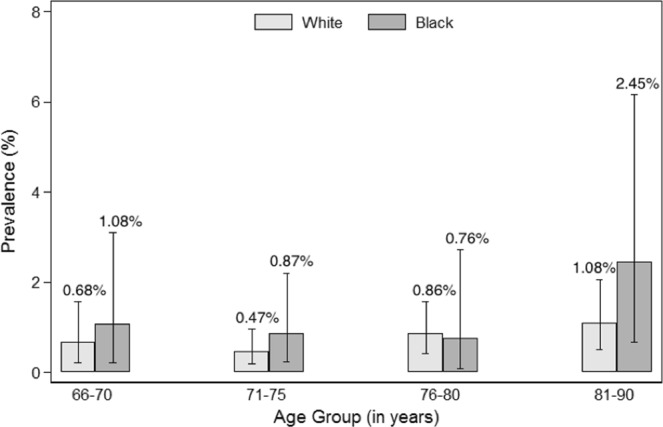
Prevalence of Overt Hypothyroidism by race and age group.

**Figure 2:**
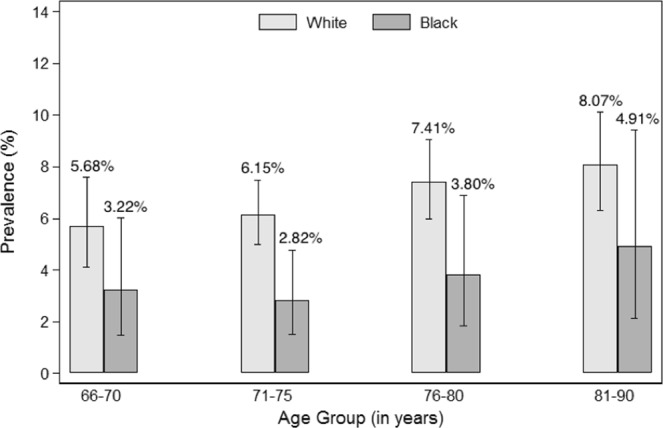
Prevalence of Subclinical Hypothyroidism by race and age group.

**Figure 3:**
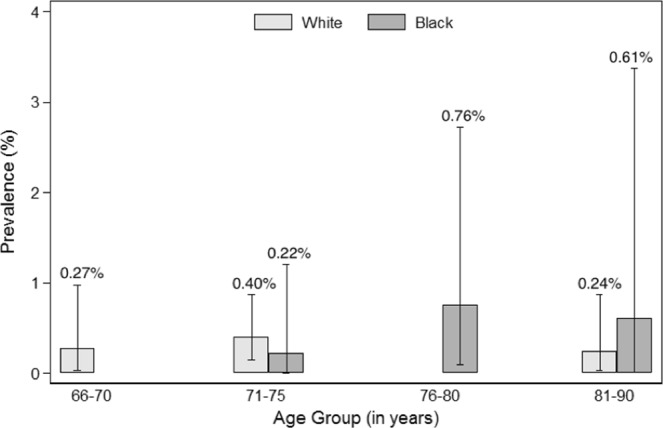
Prevalence of Overt Hyperthyroidism by race and age group.

**Figure 4:**
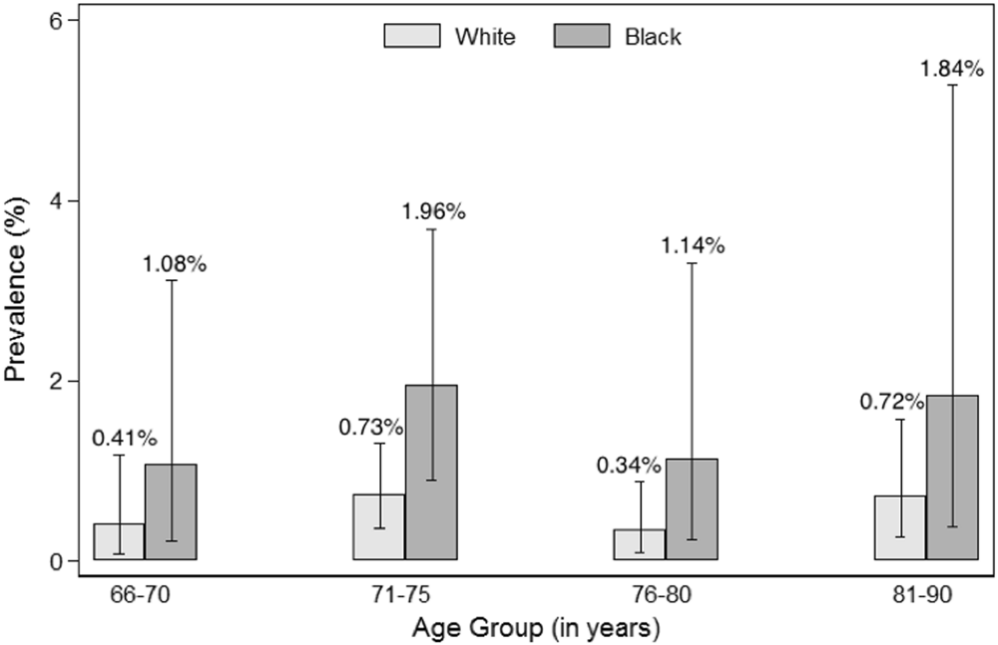
Prevalence of Subclinical Hyperthyroidism by race and age group.

Figures 1–4 show the prevalence of thyroid dysfunction categories by race and age group. Across each age group, the prevalence of subclinical hypothyroidism was higher in whites compared to blacks, with the highest prevalence found in the oldest age group (Fig. 2). The opposite trend was observed for subclinical hyperthyroidism, in which prevalence was greater in blacks compared to whites at any given age group (Fig. 4). No clear racial differences were observed for untreated overt hypothyroidism and untreated overt hyperthyroidism (Figs 1, 3).

In adjusted analyses, TSH levels in black individuals, on average, were 24.7% lower compared to whites (P < 0.001) after adjusting for sex, age, BMI, smoking and drinking history, physical activity and anti-TPO positivity (Table 2). Racial differences were also observed with FT4 and T3, in which levels were 1.32% (P = 0.023) and 3.89% (P < 0.001) lower in blacks compared to whites, respectively. Both FT4 and T3 were associated with sex, with levels 1.25% (P = 0.009) and 1.75% (P = 0.001) lower in men compared to women respectively. There were no significant independent differences in TSH by sex.

**Table 2:** Adjusted Risk Factor Associations for TSH, FT4 and T3.

	TSH, % difference (95% CI)		FT4, % difference (95% CI)		T3, % difference (95% CI)	
	Model 1*	Model 2†	Model 1*	Model 2^†^	Model 1*	Model 2^†^
Sex						
Male (Female reference)	−3.18 [−6.33, 0.07]	0.89 [−2.68, 4.59]	−0.93 [−1.78, −0.08]^C^	−1.25 [−2.17, −0.31]^C^	−2.32 [−3.28, −1.36]^C^	−1.75 [−2.80, −0.69]^C^
Race						
Black (White reference)	−25.48 [−28.37, −22.48]^C^	−24.66 [−27.88, −21.29]^C^	−1.82 [−2.82, −0.81]^C^	−1.32 [−2.44, −0.18]^C^	−2.32 [−3.46, −1.17]^C^	−3.89 [−5.14, −2.63]^C^
Age						
65–70 (Reference)	0	0	0	0	0	0
71–75	−0.21 [−4.71, 4.50]	0.51 [−4.17, 5.42]	−0.19 [−1.38, 1.00]	−0.02 [−1.25, 1.24]	−2.36 [−3.69, −1.02]^C^	−2.15 [−3.53, −0.75]^C^
76–80	4.47 [−0.57, 9.76]	3.34 [−1.84, 8.78]	−0.67 [−1.92, 0.61]	−0.53 [−1.85, 0.82]	−4.64 [−6.03, −3.24]^C^	−4.31 [−5.76, −2.83]^C^
80+	10.03 [4.26, 16.11]^C^	9.10 [3.10, 15.46]^C^	0.03 [−1.35, 1.43]	0.10 [−1.37, 1.60]	−7.39 [−8.86, −5.90]^C^	−6.58 [−8.15, −4.99]^C^
BMI (kg/m^2^)						
<25.0 (Reference)	0	0	0	0	0	0
25.0–29.9	−1.40 [−5.39, 2.77]	−1.11 [−5.23, 3.18]	−1.79 [−2.83, −0.74]^C^	−1.61 [−2.70, −0.51]^C^	2.41 [1.16, 3.66]^C^	2.31 [1.02, 3.61]^C^
30.0+	−2.29 [−6.42, 2.02]	−3.36 [−7.62, 1.10]	−1.95 [−3.04, −0.85]^C^	−1.92 [−3.07, −0.76]^C^	3.75 [2.43, 5.08]^C^	3.46 [2.08, 4.86]^C^
Smoking History						
Current Smoker (Reference)	0	0	0	0	0	0
Former Smoker	13.58 [5.92, 21.79]^C^	14.33 [6.57, 22.65]^C^	−4.50 [−6.22, −2.75]^C^	−4.26 [−6.00, −2.49]^C^	−5.40 [−7.36, −3.40]^C^	−5.97 [−7.92, −3.98]^C^
Never Smoker	20.20 [11.97, 29.05]^C^	19.55 [11.19, 28.53]^C^	−5.35 [−7.08, −3.58]^C^	−4.93 [−6.71, −3.11]^C^	−4.84 [−6.84, −2.80]^C^	−5.92 [−7.93, −3.86]^C^
Drinking History						
Current Drinker (Reference)	0	0	0	0	0	0
Former Drinker	−1.13 [−5.00, 2.90]	0.64 [−3.37, 4.81]	−0.86 [−1.88, 0.16]	−0.91 [−1.96, 0.15]	3.96 [2.74, 5.19]^C^	4.10 [2.85, 5.37]^C^
Never Drinker	6.20 [1.43, 11.19]^C^	4.28 [−0.66, 9.47]	−1.26 [−2.42, −0.08]^C^	−0.69 [−1.95, 0.57]	3.12 [1.74, 4.53]^C^	3.44 [1.96, 4.95]^C^

*Adjusted for age category, sex and race, ^†^Adjusted for variables in Model 1 plus BMI category, smoking history, drinking history, physical activity, prevalent CVD and Anti-TPO Positivity, ^C^*P* Value < 0.05.

Of all three blood tests, T3 was most strongly associated with age, with levels up to 6.6% lower in individuals older than 80 years of age compared to those ages 65–70. A clear association with age was not observed for FT4. TSH on the other hand, was positively associated with age, with levels up to 9.1% higher in individuals >80 years-old compared to 65–70 years-old (P = 0.003) (Table 2).

T3 levels were 3.5% higher in overweight or obese individuals compared to individuals with a normal BMI. By contrast, FT4 was lower at higher BMI categories. No statistically significant association was observed between TSH and BMI after adjustment.

Smoking status was significantly associated with TSH, FT4, and T3 levels. Current smokers had lower TSH levels compared to former or never smokers (P < 0.001). An inverse relationship was observed for FT4 and T3, in which current smokers had higher levels compared to former or never smokers (P < 0.001). We did not observe an association of TSH or FT4 with drinking history, although T3 levels were lower for current drinkers as compared to never or former drinkers (P < 0.001) (Table 2).

We observed sex differences in anti-TPO positivity. Men were much less likely to be auto-antibody positive compared to women [OR = 0.59 (0.47,0.75), P < 0.001]. There were no apparent racial disparities in anti-TPO positivity status [OR = 1.21 (0.93,1.57), P = 0.15]. Those ages 71–75 were less likely to be auto-antibody positive compared to those ages 65–70 [OR = 0.69 (0.52,0.91), P = 0.009]. There were no significant adjusted associations with BMI, smoking status, and drinking history (Table 3).

**Table 3:** Adjusted Odds Ratios (95% Confidence Intervals) of Anti-TPO Positivity ≥34 IU/ml.

	Model 1*		Model 2^†^	
	OR (95%CI)	*P* Value	OR (95%CI)	*P* Value
Sex				
Male (Female Reference)	0.55 [0.45,0.69]	<0.001	0.59 [0.47,0.75]	<0.001
Race				
Black (White Reference)	1.19 [0.94,1.49]	0.146	1.21 [0.93,1.57]	0.152
Age				
65–70 (Reference)	1.00	—	1.00	—
71–75	0.72 [0.55,0.94]	0.016	0.69 [0.52,0.91]	0.009
76–80	0.81 [0.61,1.08]	0.16	0.80 [0.59,1.08]	0.15
80+	0.73 [0.53,1.01]	0.059	0.73 [0.51,1.03]	0.069
BMI (Kg/m^2^)				
<25.0 (Reference)	1.00	—	1.00	—
25.0–29.9	0.88 [0.69,1.13]	0.315	0.92 [0.71,1.19]	0.52
30.0+	0.86 [0.66,1.11]	0.241	0.92 [0.70,1.22]	0.56
Smoking History				
Current Smoker (Reference)	1.00	—	1.00	—
Former Smoker	0.87 [0.57,1.32]	0.506	0.85 [0.56,1.30]	0.456
Never Smoker	0.99 [0.65,1.51]	0.955	0.91 [0.59,1.41]	0.669
Drinking History				
Current Drinker (Reference)	1.00	—	1.00	—
Former Drinker	0.93 [0.72,1.21]	0.604	0.95 [0.73,1.24]	0.73
Never Drinker	1.20 [0.92,1.56]	0.184	1.17 [0.88,1.56]	0.28

*Adjusted for age category, sex and race, ^†^Adjusted for variables in Model 1 plus BMI category, smoking history, drinking history, physical activity, and prevalent CVD.

## Discussion

In this study, we assessed the prevalence of thyroid dysfunction, and the associations of TSH, FT4, T3 levels and anti-TPO positivity with various demographic and clinical risk factors. Our study has the following important findings: (1) the prevalence of thyroid dysfunction in an older age population was nearly 25% when accounting for treated and untreated thyroid dysfunction categories, (2) significant sex and racial differences were observed in subclinical hypothyroidism, subclinical hyperthyroidism, and treated hypothyroidism in this age group, (3) significantly lower TSH, FT4 and T3 levels were observed in older males and older black individuals, and 4) thyroid hormone levels were associated with clinical risk factors including smoking history, and BMI category.

Data from the Third National Health and Nutrition Examination Survey (NHANES III) conducted from 1988–1994 in individuals aged 12 and older and representative of the general U.S. population found that approximately 4.6% had undiagnosed hypothyroidism (0.3% clinical and 4.3% subclinical) and 1.3% had undiagnosed hyperthyroidism (0.5% clinical and 0.7% subclinical). This survey also observed the prevalence of anti-TPO positivity and TSH concentrations to be greater in females, increase with age, and greater in whites and Mexican Americans compared to blacks^10^. In our study of an older population, we observed a higher prevalence of undiagnosed hypothyroidism (6.88%, 0.82% overt and 6.06% subclinical) and a smaller prevalence of undiagnosed hyperthyroidism (1.04%, 0.26% overt and 0.78% subclinical). We did observe higher levels of TSH in white participants, and men were less likely to have anti-TPO positivity compared to women. Racial disparities in thyroid hormones have been previously reported in the literature^18,21^. Although TSH levels were lower in men compared to women, our results were not significant after adjustment, and only FT4 and T3 demonstrated sex differences in older adults. One explanation for these findings could be related to hormonal action, given that women in our cohort were post-menopausal. Some studies have demonstrated that higher estrogen levels contribute to elevated levels of TSH or lower levels of FT4, resulting in hypothyroid symptoms^22,23^. In our cohort of post-menopausal women, this mechanism would be diminished.

Compared to NHANES, our estimates demonstrated a greater prevalence for all thyroid dysfunction categories (except for overt hyperthyroidism), particularly in subclinical hypothyroidism, which could partly be explained by the older population used in our analysis. Previous studies have demonstrated a higher prevalence of thyroid dysfunction in older individuals^9,24^. One study suggested that with aging, the set point for TSH secretion is altered, resulting in higher serum TSH concentrations due to diminished sensitivity of thyrotropes to negative feedback of thyroid hormones^11^. Other studies demonstrated that if age-adjusted normal ranges are used in older adults, the prevalence of thyroid dysfunction may not increase with old age and some individuals might be reclassified from “abnormal” to “normal”, avoiding unnecessary treatment^11,12^. The debate regarding age-specific cut-points for thyroid dysfunction has clinical and economic implications, given the high prevalence in older adults and decreasing TSH threshold levels for treatment over the years^25^.

The strong association between smoking and thyroid hormone levels has been previously demonstrated^17^. In a cross-sectional study of the effect of smoking on TSH levels, serum TSH levels were significantly lower in current smokers than in non-smokers^17^. Similar to these findings, we observed significantly lower levels of TSH, and higher levels of FT4 and T3 in current smokers compared to never or former smokers. Lower serum TSH levels have also been observed in active smokers in the NHANES III study^15^. While the mechanism for an association of smoking with thyroid hormone levels is unknown, one proposed explanation is that nicotine’s effect on sympathetic activation could enhance thyroid hormone secretion^17^. If causal, smoking in older adults may be an important consideration when deciding on initiating or changing thyroid hormone therapy, given that higher levels of FT4 and T3 could change treatment decisions.

Limitations of our study include the cross-sectional design, while ideal for establishing prevalence, we are unable to establish temporality of the observed risk factor associations. Furthermore, this study does not account for the general variability of thyroid function tests in an individual, and there is a possibility that some patients with transient thyroid function tests changes have been misclassified due to the cross-sectional design. As our study used a community-based population, power was limited for some analyses, especially for hyperthyroidism (n = 56 in our study population). Although we adjusted for multiple demographic and clinical risk factors in our analyses, as with any observational study, residual confounding may be a concern.

The strengths of our study include a large, well-characterized, community-based sample of older adults including a large number of blacks (21.6%), and individuals greater that 80 years-old (N = 993), which are populations that are often underrepresented. The comprehensive panel of thyroid tests and information on thyroid medications at the time of the study further adds to the strength of our analysis.

Our study demonstrated a high prevalence of thyroid dysfunction in an older, community-based population, which is higher than the previously reported prevalence of thyroid dysfunction in community-based studies with younger and middle-aged populations. Furthermore, our study demonstrated that the prevalence of undiagnosed hypothyroidism in older individuals is higher than what has previously been reported. There were clear differences by sex and race, with older women having a higher prevalence of treated hypothyroidism compared to older men, and older whites having a higher prevalence of subclinical hypothyroidism and treated hypothyroidism compared to older blacks. The prevalence of subclinical hyperthyroidism was greater in blacks compared to whites.

With rising life expectancies and aging populations, it is increasingly important to understand variations in thyroid dysfunction in older individuals and their association with risk factors encountered in daily clinical practice. Our study demonstrated a thyroid dysfunction prevalence of nearly 25% in older individuals. This high prevalence sheds a light on the potential for the underappreciation of both overt and subclinical thyroid diseases, which can have substantial effects on the management and treatment of older patients who might also present with other complex comorbidities. Our results call for greater awareness of thyroid dysfunction, and the potential need for more thyroid function screening in this age group.

## Methods

We conducted a cross-sectional study using data from the Atherosclerosis Risk in Communities (ARIC) study. This is a prospective epidemiologic cohort that began with 15,792 individuals recruited between 1987–1989 from 4 study centers in the United States (Forsyth County, North Carolina, Jackson, Mississippi, Minneapolis, Minnesota, and Washington County, Maryland)^26^. The current study involves visit 5 (2011–2013). All study participants provided informed consent at each study visit.

There were 6,538 participants, all aged 65 or older, who attended visit 5. We excluded individuals who were neither white nor African American, and those who were missing any covariate of interest. Our final study population included 5,392 participants who had complete data for all variables of interest. In regression analyses of thyroid hormone concentrations, we excluded individuals receiving current treatment for either hypothyroidism or hyperthyroidism (n = 917).

The ARIC study has been approved by the Institutional Review Boards (IRB) at all participating institutions: University of North Carolina at Chapel Hill IRB, Wake Forest University IRB, Johns Hopkins University IRB, University of Minnesota IRB and University of Mississipi Medical Center IRB. Written informed consent was obtained from all study participants. All methods were carried out in accordance with the relevant guidelines and regulations for human subject research, in accordance with the Declaration of Helsinki^26^.

### Laboratory measurements

Thyroid parameters were measured in 2012–2013 at the University of Minnesota in stored serum samples originally collected from ARIC participants at visit 5. TSH, FT4, T3 and anti-TPO were measured in serum on a Roche e411 Immunoassay Analyzer (Roche Diagnostics Corporation, Indianapolis, IN). The inter-assay coefficients of variation for the thyroid hormones were: TSH, 3.6% at a mean concentration of 0.208 mIU/L; FT4, 4.9% at a mean concentration of 1.09 ng/dL; T3, 6.7% at a mean concentration of 364.5 ng/dL; and, anti-TPO, 9.1% at a mean concentration of 133 IU/mL.

### Study variable definitions

Using ARIC defined cut-offs^18^, the following definitions for each thyroid dysfunction category were used for the main analysis as has been done in previous ARIC papers^18,27^. For comparison, we also used manufacturer (Roche) defined cut-offs to define prevalence of the various thyroid conditions. Overt hyperthyroidism: serum TSH <0.56 mlU/L and FT4 > 1.4 ng/dL (Roche, TSH <0.27 mIU/L and FT4 > 1.7 ng/dL), overt hypothyroidism: serum TSH >5.1 mlU/L and FT4 < 0.85 ng/dL (Roche, TSH >4.2 mIU/L and FT4 < 0.93 ng/dL), subclinical hyperthyroidism: normal FT4 (0.85–1.4 ng/dL) and serum TSH below lower limit of reference range < 0.56 mlU/L (TSH < 0.27 mIU/L and 0.93 ng/dL ≤ FT4 ≤ 1.7 ng/dL), subclinical hypothyroidism: normal FT4 (0.85–1.4 ng/dL) and serum TSH higher than upper limit of reference range >5.1mlU/L (TSH >4.2 mIU/L and 0.93 ng/dL ≤ FT4 ≤ 1.7 ng/dL) (Table 4). The presence of anti-TPO antibodies was defined as serum concentration ≥34 IU/ml. Treated thyroid dysfunction was defined as self-reported diagnosis by a physician and currently taking medication for the condition.

**Table 4:** Summary of ARIC and Roche cut-points for thyroid dysfunction.

	Overt Hypothyroidism	Subclinical Hypothyroidism	Overt Hyperthyroidism	Subclinical Hyperthyroidism
Roche cutoff	TSH > 4.2 mIU/L & FT4 < 0.93 ng/dL	TSH > 4.2 mIU/L & 0.93 ng/dL ≤ FT4 ≤ 1.7 ng/dL	TSH < 0.27 mIU/L & FT4 > 1.7 ng/dL	TSH < 0.27 mIU/L & 0.93 ng/dL ≤ FT4 ≤ 1.7 ng/dL
ARIC cutoff, Overall	TSH > 5.1 mIU/L & FT4 < 0.85 ng/dL	TSH > 5.1 mIU/L & 0.85 ng/dL ≤ FT4 ≤ 1.4 ng/dL	TSH < 0.56 mIU/L & FT4 > 1.4 ng/dL	TSH < 0.56 mIU/L & 0.85 ng/dL ≤ FT4 ≤ 1.4 ng/dL

BMI was calculated as kg/m^2^. Smoking history was defined as never, former, or current smoker. Alcohol use was defined as never, former, or current drinker.

### Statistical analysis

We evaluated the prevalence of thyroid dysfunction categories according to age, 65–70 (n = 1,018), 71–75 (n = 1,957), 76–80 (n = 1,424), 80 + (n = 993), sex, men (n = 2,257), women (n = 3,135), race, white (n = 4,227), black (n = 1,165), BMI category, normal weight (n = 1,387), overweight (n = 2,149), obese (n = 1,856), using ARIC defined cut-off points. P-values for differences between thyroid dysfunction categories were obtained using chi-squared analysis compared to the euthyroid group.

Distributions of continuous variables were assessed for normality using Shapiro-Wilk test and Kernel plots. Because the distribution of some continuous variables was not normal, natural log-transformation was performed to TSH, FT4, and T3, in which normality was approached after transformation.

We used multivariable linear regression to examine the association between log-transformed TSH, FT4, T3 and various demographic and clinical risk factors including sex, race, age, BMI, smoking and drinking history. Effect estimates from the multivariable linear models and their corresponding 95% confidence intervals were converted to percent change in TSH, FT4, or T3 levels for the presence of each categorical and continuous predictor variable by exponentiation of the effect estimate, subtracting 1, and multiplying by 100, i.e. 100 × (e^β^ − 1). We used logistic regression to examine the association of anti-TPO positivity and the same demographic and clinical risk factors. Odds ratios and their corresponding 95% confidence intervals were calculated.

We evaluated two models to assess for these associations after adjustment for sex, race and age (model 1) and additionally BMI, smoking history, drinking history, physical activity level, and diagnosis of cardiovascular disease (model 2) for each of TSH, FT4, T3 and anti-TPO positivity. All tests performed were two tailed and statistical significance was determined to be at P < 0.05.

All statistical analyses where performed using Stata/SE 14 (StataCorp, College Station, TX).

## Data Availability

The datasets generated and analysed during the current study are available from the corresponding author, Dr. Elizabeth Selvin (eselvin@jhu.edu), on reasonable request. Most ARIC data can be also obtained from BioLINCC, a repository maintained by the National Heart, Lung, and Blood Institute. The BioLINCC website^28^ includes detailed information about the available data and the process to obtain such data.
